# Do the Footwear Profiles and Foot-Related Problems Reported by Netball Players Differ Between Males and Females?

**DOI:** 10.1186/s40798-022-00495-y

**Published:** 2022-08-06

**Authors:** Maddison M. Kirk, Joshua P. M. Mattock, Celeste E. Coltman, Julie R. Steele

**Affiliations:** 1 Biomechanics Research Laboratory, School of Medical, Indigenous and Health Sciences, Faculty of Science, Medicine and Health, University of Wollongong, Wollongong, NSW 2522 Australia; 2 University of Canberra Research Institute for Sport and Exercise, Faculty of Health, University of Canberra, Canberra, Australia

**Keywords:** Netball, Footwear, Shoes, Performance, Injury

## Abstract

**Introduction:**

We explored the footwear profiles and foot-related problems reported by netball players and whether these differed between males and females.

**Methods:**

Two thousand nine hundred and twenty-five amateur, sub-elite and elite netball players (men *n* = 279; women *n* = 2646; age 26.4 ± 10.0 years) completed a custom-designed online survey with questions related to netball experience, current netball footwear habits and history of foot-related problems. Footwear profiles and foot-related problems were considered in logistic regressions against sex and competition level to ascertain significant relationships (*p* < 0.05) and predictive values (odds ratio).

**Results:**

Although 80.4% of respondents reported wearing netball-specific shoes, females were 13.2 times more likely to wear netball-specific shoes than males. Foot-related problems and foot pain were reported by 84.3% and 56.8% of netball players, respectively; with blisters, ankle sprain/strains and calluses being most common. Although women were significantly more likely to suffer from foot-related problems than men, males were significantly more likely to believe their foot pain was caused by the footwear they wore for netball.

**Conclusion:**

The high prevalence of foot-related problems and pain reported by all netball players suggests that the shoes players are currently wearing for netball are not meeting the requirements of players, particularly regarding fit, comfort and functionality. As male netball players have significantly different footwear profiles to female players, men are likely to require netball-specific footwear that differs to the netball-specific shoes designed for female players.

**Supplementary Information:**

The online version contains supplementary material available at 10.1186/s40798-022-00495-y.

## Key Points

- A high prevalence of foot-related problems and pain reported by all netball players suggests that the shoes players are currently wearing for netball are not meeting the requirements of players, particularly regarding fit, comfort and functionality.
- Footwear profiles and foot-related problems differ significantly between male and female netball players.
- Men are likely to require netball-specific footwear that differs to the netball-specific shoes designed for female netball players.

## Introduction

Netball is a popular court sport played by an estimated 20 million participants across 80 countries, particularly within the Commonwealth [1]. Although netball is predominantly played by females, there are a growing number of males participating in the sport. Males now play netball in 16 countries, including Australia, New Zealand, the United Kingdom, Canada, Jamaica, as well as parts of Africa, Asia and the Pacific Island Nations [2]. A recent report revealed a 44.5% and 379.4% increase in men’s and boy's participation in netball in Australia since 2016, respectively—the highest growth rate of any adult men’s sport in Australia [3, 4]. This percentage is projected to continue to rise, given that increasing male participation rates have been identified as a key strategy to achieve netball’s inclusion in the Olympic Games and to support gender equality in the sport [5].

Netball requires players to perform repeated bursts of rapid acceleration, sudden changes of direction and frequent jumping [6]. Although these skills are essential for effective netball performance, the skills place high stress on the lower extremity and, consequently, there is a high injury rate in the sport [7]. The most common site of injury in netball is to the lower limb, with ankle (53.8%) and knee (27.7%) injuries being the most frequently experienced by female community netball players [8]. More recently, foot and ankle injuries have been shown to account for 41% of all injuries sustained by female sub-elite netball players during a national netball tournament [9]. Any abnormal or erroneous movement of the foot could explain this high incidence of lower limb injuries in netball, particularly as foot biomechanics can influence more proximal joints such as the ankle, knee, hip and lower back [10, 11]. A primary factor that alters the loading and movement of the foot is footwear [12]. Athletic footwear is designed to provide traction and motion control, protect the plantar surface of the foot and attenuate impact forces generated during activity [13]. Therefore, to minimise the potential for injuries and enhance performance in netball, the shoes worn by players must suit the demands of the game [14, 15].

In the early 1900s netball-specific shoes were modelled on sandshoes, with the upper primarily made from canvas and combined with a thick rubber outsole with minimal cushioning [16]. The increasing professionalisation and popularity of netball led to substantial biomechanical research investigating netball movements performed by female players [14]. Based on this research, netball-specific shoes evolved into more sophisticated low-cut shoes that are designed to withstand the high impact forces and multi-directional movements characteristic of the sport [17]. No biomechanical research, however, has ever examined movements performed by male netball players to provide evidence upon which to develop a netball-specific shoe for men. Despite the importance of functional footwear in enhancing performance and minimising injury risk, there is a large gap in the scientific literature examining the type of shoes worn by netball players, particularly male netball players. In fact, the main research investigating footwear profiles of netball players was conducted over 35 years ago in a female-only cohort [18]. This research revealed that netball-specific shoes were not meeting the requirements of netball players, whereby 49% of players complained of having problems with their shoes and feet, including blisters, chafing and soreness [18]. Only 9% of these players, however, attributed the injuries they incurred during netball to the footwear that they wore when on the court [18].

Since the late 1980s, there have been changes to the demands and movement patterns in netball because of the introduction of new game rules and other developments in the sport [19]. There have also been numerous technological advancements in the design and methods used to manufacture netball-specific footwear. This has included introducing more durable midsole cushioning, high abrasive rubber outsoles, synthetic mesh upper materials and lateral stability systems to support feet during high-impact movements [17]. Consequently, a wide variety of netball-specific shoes now exist, allowing netball players to select a model that best suits their foot shape and style of play. Of the 18 netball-specific shoes available in Australia in April 2021 (i.e. study time-period) [20, 21], only one shoe model was marketed explicitly for men. This men’s netball-specific shoe, however, was a hybrid-court shoe, designed to suit both netball and volleyball (Personal communications, Mark Doherty; ASICS Oceania Pty Ltd, June 2019). Researchers have consistently reported that male and female feet differ in size relative to stature, as well as in shape, especially in the angle formed by the axis of the metatarsal heads and the dimensions of the arch [22, 23]. No research was located, however, comparing the foot shape of male and female netball players. To ensure shoes fit and function properly, it is imperative that any shoe last is based on the foot dimensions of individuals who are likely to wear the shoes; if not, ill-fitting footwear can lead to a greater risk of foot-related problems, pain and injury [24].

Given the evolution of footwear and netball and the lack of any published research pertaining to the footwear needs of male netball players, it is imperative that we gain a better understanding of the current footwear profiles and foot-related problems reported by both male and female netball players. This information will provide evidence to inform recommendations to improve footwear for netball, particularly for male netball players, to ensure netball-specific shoes are designed to fit and function properly and, in turn, minimise foot-related problems. Therefore, this study aimed: (1) to document the current footwear profiles and foot-related problems reported by male and female netball players and (2) to determine whether the footwear profiles and foot-related problems differed between male and female netball players. It was hypothesised that the footwear profiles and foot-related problems of male netball players would differ significantly when compared to female netball players.

## Methods

### Participants and Survey Implementation

Inclusion criteria were males and females, over 16 years of age, who had participated in at least one season of netball during the two years before the survey was released. Participants under 16 years of age and/or who completed less than 50% of the survey were excluded (so that all participants had completed at least part of the second section of the survey). Netball players satisfying the inclusion criteria were indirectly recruited to complete an online survey about their netball experience, current netball footwear habits and history of foot-related problems. A link to the online survey was distributed via email to 590 community, regional and state netball organisations across all of Australia. The link was also sent to the *Suncorp Super Netball* and *ANZ Premiership Netball* franchises, *Netball New Zealand* and *Netball Australia* and the *Australian* and *New Zealand Men’s and Mixed Netball Associations.* These organisations could then elect to share the study details and survey link with their athletes. The survey was also promoted and distributed on social media platforms and in closed international Facebook groups to athletic staff, coaches and netball players. The survey was activated in April 2020 and was closed in April 2021, when further promotion of the study failed to elicit new responses. Players were deemed to have given tacit consent to participate in the study if they completed and submitted the survey after reading the participant information sheet on the first page of the online survey. Due to the anonymity of the data collection procedures and the ‘sharing’ nature of the Internet, the survey’s response rate could not be tracked. The University of Wollongong Human Research Ethics Committee (HREC 2019/326) approved the survey design and all implementation procedures.

### Survey Design and Content

Following a review of the literature and a series of meetings and emails with netball shoe manufacturers, an anonymous 38-question online survey was designed to gather data on footwear worn by male and female netball players. The survey was comprised of four sections (About You, About the Shoes You Wear During Netball, About Your Ideal Netball Shoe and About Your Foot Comfort) and included 32 multiple-choice questions and 6 open-ended questions. Before implementing the survey, it was reviewed by three focus groups comprised of male and female netball players (*n* = 30) to evaluate the readability, content and face validity of the survey. The survey was then modified based on feedback from the focus groups before a final version of the survey was tested for reliability. The survey was published on a University of Wollongong Qualtrics account (v0217; Provo, UT), where responses were also housed. Duplicate responses were removed by firstly checking Internet protocol addresses embedded in the responses and secondly by reviewing each respondent’s characteristics and netball experience such as age, shoe size and level of competition. All returned survey responses were then exported into Microsoft Excel for analysis. Only survey items relevant to the aims of the current study are presented within this paper (see Additional file 1: Appendix A).

### Analytical Variables

#### Participant Characteristics

Participants were asked to report which netball code(s) they currently played, with response options being “Women’s Netball”, “Men’s Netball” and/or “Mixed Netball”. Respondents who selected “Mixed Netball” were asked to report whether they identified as a male or female netball player. Participants were then grouped as either male or female, which is how players are grouped within netball. These categories also reflect the way footwear is currently marketed to netball players. Respondents then reported which country they were competing in for netball, with response options being “Australia”, “New Zealand”, “United Kingdom”, “South Africa” and “Other”. Participants were asked to write their age in years and were then classified into the age groups: “16–24”, “25–34”, “35–44”, “45–54”, “55–64” and “65+”. Participants recorded the highest level of netball that they were currently competing in. This response was then used to classify each participant into one of three grading categories: (1) “Amateur”—in which players competed at a community or recreational level, (2) “Sub-elite”—in which players competed at a regional or state level and (3) “Elite”—in which players competed at a national or international level. Finally, participants were asked to select two positions that they predominantly played in netball. Based on their response the players were grouped into one of four positional groups: (1) “Attack”—Goal Shooter (GS)/Goal Attack (GA), GA/Wing Attack (WA) or GS/WA, (2) “Mid-court”—WA/Centre (C), WA/Wing Defence (WD) or WD/C, (3) “Defence”—Goal Defence (GD)/Goal Keeper (GK), GD/WD or GK/WD and (4) “Other”—any other positional combination that did not fall within the other three categories (e.g. GS/C).

#### Footwear Profiles

Participants were asked to report the brand and type of shoe they predominantly wore during their current netball activity with closed-ended response categories. Response options for footwear brand were “*ASICS*”, “*Mizuno*”, “*Nike*”, “*Adidas*”, “*Brooks*”, “*Puma*”, “*Reebok*” and “*Other*”. For footwear type, the response options were “Netball”, “Running”, “Basketball”, “Volleyball”, “Tennis”, “Cross-trainer” and “Other”. Participants were also asked “*Do you currently wear a shoe specifically manufactured for netball during your netball activity?*”, to which they could respond either “yes” or “no”. Male respondents who answered “yes” were prompted with a follow-up question asking whether they had ever worn a netball shoe specifically marketed for females. Reporting on the pair of shoes that they predominantly wore during netball, players were asked “*Do you wear this pair of shoes for activities other than netball?*”, to which they could respond either “yes” or “no”.

#### Foot-Related Problems

Foot-related problems were defined by a close-ended question where participants selected any foot-related problem(s) they had experienced during the last 12 months that were caused by their netball activity. Participants selected “no” if they did not experience any foot-related problems. Participants were then asked whether they had ever experienced foot pain caused by their netball activity, “yes” or “no”. Those players who answered “yes” were prompted with a follow-up question asking whether they currently experienced this pain during and/or after netball activity. Players who answered “yes” were asked to elaborate with close-ended questions regarding frequency of pain on a five-point Likert scale (1 ‘rarely’ to 5 ‘always’) and selecting “yes” or “no” as to whether they believed this pain was related to the footwear they wore during netball and/or had an impact on their netball performance.

### Statistical Analysis

#### Descriptive Statistics

Responses to the survey items were coded and counted to determine the frequency of each possible response. The number of responses for each question differed from the overall number of survey participants due to non-responses, multiple answer selection or when questions were only displayed if a specific condition was met. The response rate of participants completing the entire survey was 83.6%. If a participant did not complete a question, the blank response was treated as “N/A” and removed from the database for that question only.

#### Group Differences

Chi-squared tests of homogeneity were applied to each participant characteristic variable (age, country of competition, competition level and playing position), with the characteristic set as the independent variable and sex (male and female) set as the dependent variable. Post hoc analysis involved pairwise comparisons (*p* < 0.05) using the z-test of two proportions with a Bonferroni correction, to determine where any significant differences lay. The purpose of this statistical design was to determine whether any participant characteristic differed significantly between the male and female respondents and, in turn, would need to be controlled for in the logistic regressions.

#### Relationship Analysis

The footwear profile and foot-related problem data were analysed using a logistic regression design to control for sex (male and female) and competition level. For competition level, netball players who competed at a sub-elite and elite level were classified as “representative” and netball players who competed at an amateur level were deemed “recreational”. These competition level groups were combined so that there were a similar number of cases in each cell, required for the binary regressions. Footwear type and brand were analysed using multinomial logistic regression. Each footwear profile was set as the dependent variable against the independent variables of sex (male vs female) and competition level (recreational vs representative). The likelihood of netball players wearing netball-specific shoes and experiencing foot-related problems and foot pain were assessed using binary logistic regression. Whether a participant wore a netball-specific shoe (yes vs no) was inserted as a dependent variable against the independent variables of sex and competition level. The same process was repeated for the occurrence of foot-related problems and foot pain and whether this pain was caused by footwear and/or impacted sporting performance set as the dependent variable. The alpha level was set at *p* < 0.05 and all statistical analyses were conducted using IBM SPSS Statistics (Version 27.0, USA).

## Results

**Fig. 1:**
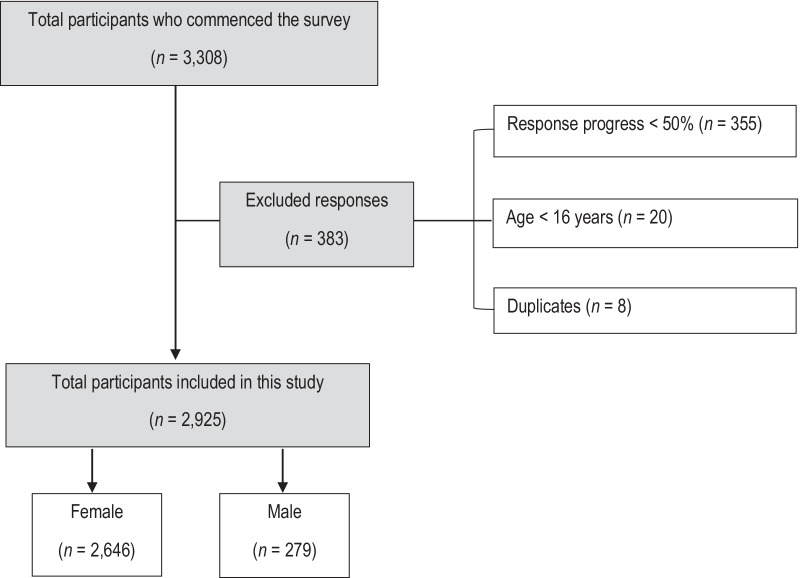
Flowchart representing the exclusion criteria and the total number of participants included in the study

The number of surveys included and excluded in the study and the reasons for exclusion are listed in Fig. 1.

### Participant Characteristics

Participant characteristics, grouped by sex, are summarised in Table 1. Participants were aged 16–71 years with a mean shoe size of 9.2 ± 2.2 US sizing (males = 13.4 ± 2.7, females = 8.8 ± 1.6). Although there were substantially more female respondents than males, this uneven distribution of responses (9.5% male vs 90.5% female) reflects the proportion of each sex in netball in Australia during the survey time-period (males 13.2%; females 86.8%; [25]). There was a significant difference between the males and females for the variables age, country of competition and competition level (see Table 1). Of these variables, post-hoc analysis revealed there were significant differences between the sexes in all competition levels, where significantly more of the males participated at an elite level and significantly less of the males participated at an amateur level, compared to females. Men’s netball is currently an emerging sport. It is therefore not surprising that a greater proportion of the men were participating at a national or international level relative to female netball players. Although statistically significant overall, post-hoc analyses revealed that the differences between the sexes in the number of participants in the age and country of competition categories were not significant across all sub-categories of these variables. Therefore, only competition level was controlled for in the logistic regressions.

**Table 1:** Characteristics of the male and female netball players who completed the survey and associations between each characteristic variable and sex

	Total (*n* = 2925)	Male (*n* = 279)	Female (*n* = 2646)	*x*^2^	*p* value
	*n* (%)	*n* (%)	*n* (%)		
*Age (years)*					
16–24	1574 (53.8)	96 (34.4)	1478 (55.9)	46.72	< 0.001*
25–34	763 (26.1)	115 (41.2)	648 (24.5)	36.63	< 0.001*
35–44	395 (13.5)	51 (18.3)	344 (13.0)	6.02	0.014
45–54	150 (5.1)	13 (4.7)	137 (5.2)	0.14	0.709
55–64	36 (1.2)	4 (1.4)	32 (1.2)	0.1	0.747
65+	7 (0.2)	0 (0)	7 (0.3)	0.74	0.39
*Country of competition*					
Australia	2327 (79.6)	240 (86.0)	2087 (78.9)	7.93	0.005*
United Kingdom	415 (14.2)	3 (1.1)	412 (15.6)	43.56	< 0.001*
New Zealand	99 (3.4)	20 (7.2)	79 (3.0)	13.5	< 0.001*
Other	51 (1.7)	13 (4.7)	38 (1.4)	15.31	< 0.001*
South Africa	33 (1.1)	3 (1.1)	30 (1.1)	0.01	0.93
*Competition level*					
Amateur	1529 (52.3)	44 (15.8)	1485 (56.1)	165.64	< 0.001*
Sub-elite	1052 (36.0)	48 (17.2)	1004 (37.9)	47.32	< 0.001*
Elite	332 (11.4)	186 (66.7)	146 (5.5)	937.76	< 0.001*
Not specified	12 (0.4)	1 (0.4)	11 (0.4)	–	–
*Playing position*					
Attack	860 (29.4)	91 (32.6)	769 (29.1)	1.54	0.215
Defence	807 (27.6)	81 (29.0)	726 (27.4)	0.32	0.571
Mid-court	746 (25.5)	68 (24.4)	678 (25.6)	0.21	0.648
Other	512 (17.5)	39 (14.0)	473 (17.9)	2.66	0.103

Bonferroni adjusted alpha level = *p* < 0.05 ÷ number of comparisons

Age = *p* < 0.0083

Country of competition = *p* < 0.01

Competition level and playing position = *p* < 0.0125

*Indicates a significant difference

### Footwear Profiles

The frequency and percentage of responses and odds ratios characterising the footwear profiles of the male and female respondents are shown in Table 2 and Table 3. During the logistic regression analysis, the categories “*Reebok*” and *“Puma”* were excluded because the variables had too few responses (*n* = 5). The category “*Other*” was also excluded because it was heterogeneous and gave implausible odds (*n* = 62). Sex was identified as a significant predictor of the shoe type and shoe brand worn by netball players, whereby males were significantly more likely to report wearing running, basketball, volleyball, tennis or cross-trainers and the brands *Nike*, *Adidas* and *Brooks,* compared to the females. Of the total respondents, 29.1% of participants reported wearing more than one pair of shoes for their netball activity, with females 42.9% less likely to wear their shoes for other activities than males (see Table 3). When responding to the question “*Do you wear a shoe specifically manufactured for netball during your netball activity*?”, 80.4% of the participants answered “yes”. Sex was also significantly related to the likelihood that participants wore netball-specific shoes, with females 13.2 times more likely to wear netball-specific shoes compared to males (see Table 3). Of the men who reported wearing netball-specific shoes, 27.4% of them indicated that they had previously worn netball-specific shoes that were specifically marketed for females.

**Table 2:** Frequency of responses and multinomial logistic regression values (with odds ratio) for predicting the footwear type and brand based on sex, while controlling for competition level

	Total (*n* = 2925)	Male (*n* = 279)	Female (*n* = 2646)	Odds ratio (95% CI)	*p* value
	*n* (%)	*n* (%)	*n* (%)		
*Shoe type*					
Netball	2278 (78.9)	83 (30.0)	2195 (84.1)	–	–
Running	354 (12.3)	50 (18.1)	304 (11.6)	4.70 (3.20–6.92)	< 0.001**
Basketball	97 (3.4)	90 (32.5)	7 (0.3)	287.57 (125.53–658.78)	< 0.001**
Cross-trainer	89 (3.1)	29 (10.5)	60 (2.3)	16.27 (9.48–27.92)	< 0.001**
Tennis	34 (1.2)	17 (6.1)	17 (0.7)	17.77 (8.53–37.04)	< 0.001**
Other	29 (1.0)	3 (1.1)	26 (1.0)	–	–
Volleyball	7 (0.2)	5 (1.8)	2 (0.1)	48.45 (8.43–278.65)	< 0.001**
Not specified	37 (0.0)	2 (0.0)	35 (0.0)	–	–
*Shoe brand*					
ASICS	2444 (84.0)	143 (51.8)	2301 (87.4)	–	–
Mizuno	171 (5.9)	8 (2.9)	163 (6.2)	0.67 (0.32–1.40)	0.283
Nike	160 (5.5)	87 (31.5)	73 (2.8)	21.22 (14.21–31.69)	< 0.001**
Other	62 (2.1)	22 (8.0)	40 (1.5)	–	–
Adidas	45 (1.5)	12 (4.3)	33 (1.3)	5.86 (2.81–12.24)	< 0.001**
Brooks	22 (0.8)	4 (1.4)	18 (0.7)	4.96 (1.51–16.30)	0.008*
Reebok	5 (0.2)	0 (0.0)	5 (0.2)	–	–
Puma	0 (0.0)	0 (0.0)	0 (0.0)	–	–
Not specified	16 (0.0)	3 (0.0)	13 (0.0)	–	–

The most frequent category of each independent variable “Netball” and “ASICS” were set as the reference category

*Indicates a significant difference at *p* < 0.05

**Indicates a significant difference at *p* < 0.001

**Table 3:** Frequency of responses and binary logistic regression values (with odds ratio) for predicting the likelihood of players wearing netball-specific shoes and their footwear for other activities, experiencing foot-related problems and foot pain and whether their foot pain was caused by the shoes they wore during netball and/or had any impact on their sporting performance, based on sex while controlling for competition level

	Total	Male	Female	Odds ratio (95% CI)	*p* value
	*n* (%)	*n* (%)	*n* (%)		
*Wear shoes for other activities than netball*					
Yes	1065 (36.4)	124 (44.4)	941 (35.6)	0.57^a^	< 0.001**
No	1860 (63.6)	155 (55.6)	1705 (64.4)	(0.44–0.74)	
*Netball-specific shoe wear*					
Yes	2325 (80.4)	89 (32.6)	2236 (85.4)	13.22	< 0.001**
No	566 (19.6)	184 (67.4)	382 (14.6)	(9.87–17.7)	
Not specified	34 (0.0)	6 (0.0)	28 (0.0)		
*Foot-related problems*					
Yes	2081 (84.3)	204 (81.6)	1877 (84.5)	1.5	0.028*
No	389 (15.7)	46 (18.4)	343 (15.5)	(1.05–2.14)	
Not specified	455 (0.0)	29 (0.0)	426 (0.0)		
*Foot pain*					
Yes	1185 (56.8)	114 (54.3)	1071 (57.0)	1.08	0.612
No	903 (43.2)	96 (45.7)	807 (43.0)	(0.80–1.45)	
*Foot pain is caused by the shoes worn during netball*					
Yes	740 (57.7)	98 (67.6)	642 (56.4)	0.61^a^	0.010*
No	543 (42.3)	47 (32.4)	496 (43.6)	(0.42–0.89)	
*Foot pain has impacted performance*					
Yes	466 (63.4)	64 (65.3)	402 (63.1)	0.87^a^	0.567
No	269 (36.6)	34 (34.7)	235 (36.9)	(0.55–1.39)	

The first category of each independent variable “Yes” and “Males” (for “Sex”) was set as the reference category

*Indicates a significant difference at *p* < 0.05

**Indicates a significant difference at *p* < 0.001

^a^Odds ratio values that were < 1.00, 1.00 was subtracted from the value and expressed as a percentage i.e. (0.57–1.00) * 100 = 42.9%; (0.61–1.00) * 100 = 39.3% and (0.87–1.00) * 100 = 13%

### Foot-Related Problems

**Fig. 2:**
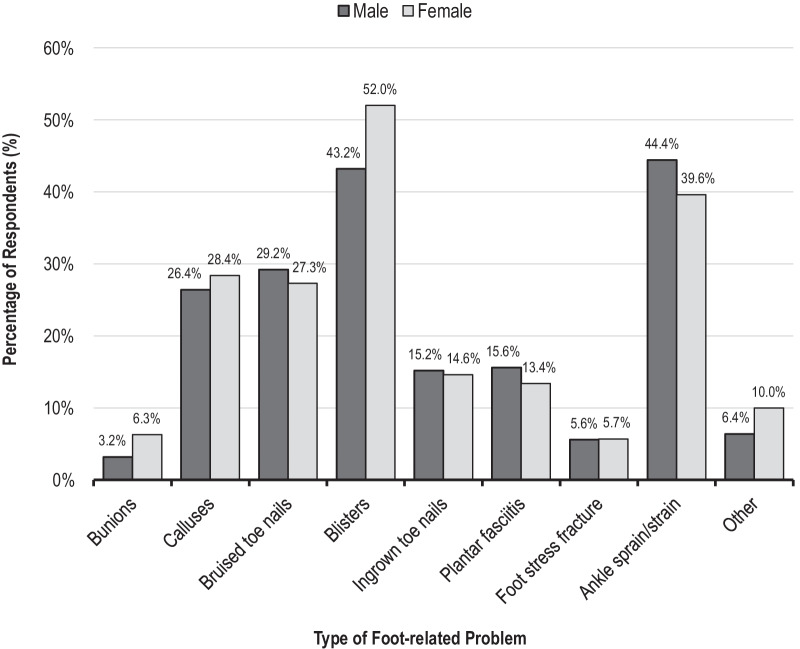
Foot-related problems that male and female respondents (male *n* = 250; female *n* = 2220) reported were caused by netball activity during the last 12 months

Foot-related problems were reported by 84.3% of participants. Ankle sprain/strains, blisters and bruised toenails were the most common problems reported by male netball players, whereas the most common problems reported by females were blisters, ankle sprains/strains and calluses (see Fig. 2). Sex was significantly related to whether a netball player reported experiencing foot-related problems, whereby females were 1.5 times more likely to suffer from foot-related problems compared to males (see Table 3). Of the participants who reported currently experiencing foot pain, 42.5% said that the foot pain occurred “occasionally”, over half believed their foot pain was caused by the shoes they wore during netball and nearly two-thirds believed the pain was having an impact on their sporting performance. Although sex was not significantly related to whether a netball player reported experiencing foot pain or impacted performance, females were 39.3% less likely to believe that their foot pain was caused by their footwear compared to males (see Table 3).

## Discussion

Previous research examining footwear profiles and foot-related problems reported by netball players were conducted more than 30 years ago, in a female-only cohort [18]. With the recent increase in males participating in netball, as well as changes to the sport and footwear over time it was imperative to investigate what footwear netball players were wearing and whether footwear profiles and foot-related problems differed between sexes. The findings of our unique study established substantial differences in the footwear profiles of male and female netball players. Importantly, foot-related problems and pain were prevalent across all netball players, and the footwear profiles of male netball players differed relative to their female counterparts. The implications of these novel findings with respect to improving netball-specific footwear are discussed below.

Netball players in the present study reported wearing a diverse range of footwear types and brands when participating in netball (see Table 2). Although most participants (84%) indicated that they wore *ASICS* footwear during netball, Hopper et al. [18] reported that *Adidas* and *Dunlop* dominated 61% of the netball footwear market over three decades ago when the researchers surveyed 420 female representative netball players. The manufacturers of netball footwear have therefore changed greatly as netball has evolved as a sport, with *ASICS* and *Mizuno* being the current leading manufacturers of netball-specific shoes worldwide. Interestingly, males were significantly more likely to report wearing *Nike*, *Adidas* and *Brooks* shoe brands when playing netball, whereas a larger proportion of females reported wearing *ASICS* and *Mizuno* shoes for netball. This difference in shoe brand preference is likely because many of the male respondents reported that they preferred to wear other shoe types, in particular basketball and running shoes, with females 13.2 times more likely to wear netball-specific shoes compared to males (see Table 3). Brands such as *Nike*, are also known to be more popular among male athletes, with women’s products accounting for only 24% of the company’s revenue in 2021 [26]. This brand bias could possibly contribute to the differences between the male and female netball players in the choice of footwear brand. The large proportion of netball players, particularly men, who reported not wearing a shoe specifically designed for netball is alarming given that men’s netball is an emerging sport, and a high proportion of males are currently participating at the elite level. It is important that this problem is addressed because players must wear shoes that have been designed to cater for the demands of the game to minimise injuries and enhance their performance [14, 15].

Unlike running, netball is a game that demands rapid acceleration to ‘break free’ from an opponent, sudden and explosive changes in direction combined with elevating leaps to receive a high pass, intercept a ball or rebound after an attempted goal [6]. Sinclair et al. [15] investigated the effects of different footwear (netball-specific shoes—*ASICS* Gel-Netburner Professional 9 versus minimalist shoes—*Nike* Free run 5.0+) on the kinetics and kinematics of three netball specific movements (run, 45° cut and jump) performed by 12 experienced female netball players. Based on the study results, the researchers cautioned that netball players should not wear shoes designed for running during netball activities [15]. Minimalist shoes were shown to be associated with significant increases in impact-loading (average loading rate) for all three netball-specific movements, as well as increases in peak ankle joint eversion during the running condition, which may increase the risk of injury [15]. Instead, the researchers recommended players should use netball-specific footwear, which has additional midsole cushioning and a more pronounced medial support mechanism designed to control excessive ankle eversion [15]. Similarly, basketball also poses different demands than netball. That is, while basketball players can dribble the ball down a court, upon catching a ball netball players must not let their landing foot be re-grounded if it is lifted while they are in possession of the ball. That is, netball players are restricted to taking a maximum of one-and-half steps in any direction while holding the ball [19]. Netball footwork rules, therefore, greatly restrict the distance over which a player may move and, in turn, reduce the time over which a player’s forward progression can be halted [6]. Stopping rapidly after receiving a pass in netball is achieved by applying an opposing horizontal frictional force; the greater the desired deceleration the larger the frictional force must be [27]. Unlike basketball shoes, netball-specific shoes are designed with increased traction to withstand the frictional forces associated with the stop-start nature of netball and the harder outdoor netball playing surfaces such as asphalt. The reasons why netball players choose not to wear netball-specific shoes and instead prefer to wear basketball and running shoes remains unclear. This may in part be due to the lack of a men’s netball-specific shoe being available or a lack of knowledge of the importance of appropriate footwear to optimising netball performance and minimising the risk of injury [14, 15]. Not wearing netball-specific shoes also implies that netball players may be unsatisfied with the current netball-specific footwear on the market, although further research is required to confirm or refute this notion.

Irrespective of whether footwear is designed correctly for a given sport, shoes must also properly fit the feet of athletes if the shoes are to achieve their desired function. To ensure shoes cater for foot shape and are comfortable to wear, it is imperative that the last upon which a shoe is constructed is based on the foot dimensions of individuals who are likely to wear the shoes [28]. Therefore, sex differences in foot shape must be considered. On average, men have longer and broader feet compared to women. The feet of men and women also differ in shape, especially in the angle formed by the axis of the metatarsal heads and the dimensions of the arch [22, 23, 28]. This size-difference in feet was supported by our findings, whereby male netball players reported wearing larger shoe sizes compared to the females (13.4 ± 2.7 vs 8.8 ± 1.6 US sizing, respectively). As male and female feet differ in size relative to stature and in shape [22, 23], it is concerning that 27.4% of male netball players indicated that they have previously worn netball-specific shoes that were specifically marketed for females. This is potentially problematic because footwear that is designed using foot dimensions unrelated to the individuals who are likely to wear the shoes can lead to ill-fitting footwear and, in turn, can lead to pain, discomfort and greater risk of foot-related problems and injury [24]. Developing netball-specific footwear for males based on the foot dimensions of men is necessary to ensure proper shoe fit and to prevent foot-related problems.

Foot-related problems such as blisters, ankle sprains, calluses and bruised toenails were very common amongst this cohort of netball players (see Fig. 2), with 84.3% of netball players reporting they suffered from at least one foot-related problem during their current netball season (see Table 3). These findings are substantially higher than previous research in which 47.5% of netball players reported experiencing problems with their feet, including 42% who required extra foot protection such as band-aids or an extra pair of socks [18]. Similarly, more recent research from Smyth et al. [9] found that lateral ankle ligament sprains and foot blisters had the highest incidence across injuries requiring medical attention during both the U17 and U19 age divisions at a national netball competition. Interestingly, in our survey females were 1.5 times more likely to suffer from foot-related problems compared to males (see Table 3). The reasons why females were more likely to suffer from foot-related problems is unclear and warrants further investigation. However, nearly 85% of netball players in the present study reported that they have previously experienced foot pain caused by their netball activity and 56.8% of these respondents were currently experiencing this pain during and/or after netball. Interestingly, sex was not related to the reporting of foot pain, whereby a large percentage of both male and female netball players reported experiencing foot pain caused by netball activity (see Table 3). Overall, the high prevalence of foot-related problems and pain reported by all netball players suggests that the shoes players are currently wearing for netball are not meeting the requirements of players, particularly regarding fit, comfort and functionality. Further research is therefore warranted to determine what factors associated with the shoes worn by both male and female netball players are contributing to this high prevalence of foot problems and pain.

Pain anywhere in the body has the potential to negatively affect muscle activity and, in turn, performance [29, 30]. Of concern, 57.7% of respondents who currently suffered from foot pain believed their pain was partially caused by the footwear they wore during netball activity, with 63.4% of these respondents reporting that their foot pain impacted their sporting performance. This percentage is much higher than three decades ago, where only 9% of the female players attributed their foot problems to their footwear [18], despite recent advancements in footwear design and methods used to manufacture netball-specific footwear. Importantly, males were significantly more likely to believe their foot pain was caused by the shoes that they wore for netball compared to females. This finding may be a consequence of male netball players wearing ill-fitting footwear and/or not wearing netball-specific shoes during their netball activity, although further research is required to confirm or refute this notion. As more men are not wearing netball-specific shoes, it is possible that men are more likely to blame their non-netball-specific footwear for their foot-related problems and pain due to how footwear type is marketed as a tool to prevent these problems in netball. Irrespective of the reason, it is vital that foot-related problems and pain are prevented so that the subsequent impacts on performance are minimised, particularly as maximising performance is necessary to increasing the professionalisation of netball for men.

### Limitations

As the results of the present study are based on data collected from a survey, it is necessary to acknowledge the inherent limitations associated with subjective, self-reported data (e.g. frequency of foot pain) such as recall bias. Participant knowledge and personal beliefs must also be considered as a limitation when responding to questions related to footwear type and whether participants believe their shoes are causing their foot-related problems and pain. It is also important to acknowledge the multi-factorial nature of foot-related problems and pain, although many respondents reported that their netball activity caused these problems, there may be other factors that could contribute to the development of these problems (e.g. familial and biomechanical factors related to bunion development) [31]. Lack of questions related to training frequency must also be considered a limitation; it may be that male netball players train less frequently compared to their female counterparts at an equivalent level, which could influence the prevalence of foot-related problems and pain. The use of an online survey and indirect recruitment methods also prevented us from calculating an accurate response rate. A limited number of responses for certain categories, such as volleyball and basketball shoe types, are acknowledged, and future research that includes more responses in these categories is recommended to derive more accurate odds ratios. It is also important to acknowledge that the survey was distributed during the COVID-19 pandemic, which could have potentially impacted the recruitment process and influenced the participants responses, given that most netball competitions were abandoned in 2020.

## Conclusion

Irrespective of sex, the netball players in this study reported a high prevalence of foot-related problems, such as blisters, ankle sprain/strains and foot pain, suggesting that the shoes players are currently wearing for netball are not meeting the requirements of players, particularly regarding fit, comfort and functionality. Although netball players wear a diverse range of footwear, male netball players have significantly different footwear profiles relative to their female counterparts. Male netball players therefore appear to require netball-specific footwear that differs to the netball-specific shoes designed for female players. However, to improve and develop effective footwear for all netball players, further research is required to better understand player perceptions and satisfaction with the fit, comfort and functionality of the shoes they wear for netball.

## Supplementary Information

**Additional file 1**. The survey items included in the study.

## Data Availability

All data generated or analysed during this study are included in this published article.
