# Supplementary material for: Do the Footwear Profiles and Foot-Related Problems Reported by Netball Players Differ Between Males and Females?
Source: Sports Med Open. 2022 Aug 6;8:103. doi: 10.1186/s40798-022-00495-y (PMC9357246; doi:10.1186/s40798-022-00495-y)
Supplement: Supplementary file 1 — Additional file 1. The survey items included in the study. [file 40798_2022_495_MOESM1_ESM.pdf]

Improving footwear for netball players

Start of Block: Default Question Block

Improving footwear for netball players
Despite the importance of wearing appropriate footwear to enhance performance and minimise injury risk in netball, three-quarters of netball players do not wear the correct shoes for the sport.

In this survey we aim to assess the perceptions of netball players on the design and performance of current netball footwear in order to identify potential netball shoe design features that affect shoe fit and comfort. If you choose to participate, you will answer questions about your footwear habits, any foot pain and/or lower limb injury you have experienced, and what you like and dislike about the shoes you have worn for netball in the **last 12 months**.

To complete this on-line survey, you must be: 16 years of age or older and participated in at least 1 season of representative netball in the last 2 years. It will take approximately **10 minutes** to complete the survey.

This survey is being conducted as part of a PhD thesis by Maddison Kirk at the University of Wollongong. For more information please find attached a Participant Information Sheet: [Participant information sheet]

By clicking on the button below, you consent to participate in this survey.

Page Break

I. About you
In this section we want to learn about you and your involvement with netball.

What netball have you played during the **last 12 months**? Please select all that apply.

▢ Women's netball (1)

▢ Men's netball (2)

▢ Mixed netball (3)

Display This Question:

If What netball have you played during the last 12 months? Please select all that apply. = Mixed netball

Sex:

○ Male (1)

○ Female (2)

Please write your **age** (years):

____

What **country** have you primarily competed netball in during the **last 12 months**?

○ Australia (1)

○ New Zealand (2)

○ England (3)

○ South Africa (4)

○ Other: (5) ____

Display This Question:

If What netball have you played during the last 12 months? Please select all that apply. = Women's netball

And What country have you primarily competed netball in during the last 12 months? != Australia

What is the **highest level** of Women's netball that you have competed in during the **last 12 months**?

____

Display This Question:

If What netball have you played during the last 12 months? Please select all that apply. = Men's netball

And What country have you primarily competed netball in during the last 12 months? != Australia

What is the highest level of Men's netball that you have competed in during the **last 12 months**?

____

Display This Question:

If What netball have you played during the last 12 months? Please select all that apply. = Mixed netball

And What country have you primarily competed netball in during the last 12 months? != Australia

What is the highest level of Mixed netball that you have competed in during the **last 12 months**?

____

Display This Question:

If What netball have you played during the last 12 months? Please select all that apply. = Women's netball

And What country have you primarily competed netball in during the last 12 months? = Australia

What is the **highest level** of Women's netball that you have competed in during the **last 12 months**?

- ◯ Social/club (1)
- ◯ Metro/State League (2)
- ◯ Premier League (3)
- ◯ Australian Netball League (4)
- ◯ Suncorp Super Netball (5)
- ◯ International (6)

Display This Question:

If What netball have you played during the last 12 months? Please select all that apply. = Men's netball

And What country have you primarily competed netball in during the last 12 months? = Australia

What is the **highest level** of Men's netball that you have competed in during the **last 12 months**?

- ◯ Social/club (1)
- ◯ Men's League (2)
- ◯ State team (3)
- ◯ International (4)

Display This Question:

If What netball have you played during the last 12 months? Please select all that apply. = Mixed netball

And What country have you primarily competed netball in during the last 12 months? = Australia

What is the **highest level** of Mixed netball that you have competed in during the **last 12 months**?

- Social/club (1)
- Men's League (2)
- State team (3)
- International (4)

---

*

What two **positions** have you predominantly played in netball during the **last 12 months**? Please select two options.

- GS (1)
- GA (2)
- WA (3)
- C (4)
- WD (5)
- GD (6)
- GK (7)

II. About the shoes you wear during netball
In this section we want to learn about the shoes you wear during netball and what you like/dislike about them.

What is your shoe size for the shoes you **currently** wear for netball (US sizing)? Please select at least one size from one column.

	**Women's**	**Men's**
**Shoe size** (1)	▼ 5 (1 ... 15 (15)	▼ 5 (1 ... 20 (20)

Do you **currently** wear a shoe **specifically manufactured for netball** during your netball activity? (e.g. Asics Netburner)

◯ Yes (1)

◯ No (2)

◯ I don't know (3)

Display This Question:

If What netball have you played during the last 12 months? Please select all that apply. = Men's netball

Or Sex: = Male

Have you ever worn a **netball shoe specifically marketed for females** when playing netball?

○ Yes (1)

○ No (2)

○ I don't know (3)

What **shoe/s** do you **currently** wear during netball activity? If you wear more than one pair, please list both shoes.

	**Brand**	**Category**
**Shoe 1** (1)	▼ Asics (1 ... Other (8)	▼ Netball (1 ... Other (7)

Please answer the remaining questions in this section on the <u>ONE</u> shoe that you wear the most during netball:

Do you wear this pair of shoes for any **other activities** other than netball?

○ Yes (1)

○ No (2)

IV. About your foot comfort
In this section, we want to learn about any foot pain and/or lower limb injury that you experience.

In the **last 12 months**, have you had any of the following **foot problems** that are caused by your netball activity? Please select all that apply.

- ☐ No (1)
- ☐ Bunions (2)
- ☐ Calluses (3)
- ☐ Bruised toe nails (4)
- ☐ Blisters (5)
- ☐ Ingrown toe nails (6)
- ☐ Plantar fasciitis (7)
- ☐ Foot stress fracture (8)
- ☐ Ankle sprain/strain (9)
- ☐ Other: (10) ____

Have you **ever** experienced **foot pain** caused by your netball activity?

- ○ Yes (1)
- ○ No (2)

Display This Question:

If Have you ever experienced foot pain caused by your netball activity? = Yes

Do you **currently** get this **foot pain** during and/or after your netball activity?

- Yes (1)
- No (2)

Display This Question:

If Do you currently get this foot pain during and/or after your netball activity? = Yes

How **often** do you get this foot pain?

- Rarely (2)
- Occasionally (3)
- Often (4)
- Very often (5)
- Always (6)

Display This Question:

If Have you ever experienced foot pain caused by your netball activity? = Yes

Do you believe this foot pain is **partially caused by the shoes** you wear during netball?

- Yes (1)
- No (2)
- I don't know (3)

Display This Question:

If Do you believe this foot pain is partially caused by the shoes you wear during netball? = Yes

Do you believe this foot pain caused by the shoes you wear during netball is **affecting your sporting performance**?

○ Yes (1)

○ No (2)
